# {2,2′-[4-Chloro-5-methyl-*o*-phenyl­enebis(nitrilo­methyl­idyne)]diphenolato}nickel(II)

**DOI:** 10.1107/S1600536810046088

**Published:** 2010-11-13

**Authors:** Haixia Wang

**Affiliations:** a Department of Chemistry and Environmental Science, Henan Normal University, Xinxiang 453007, People’s Republic of China

## Abstract

In the title complex, [Ni(C_21_H_15_ClN_2_O_2_)], the Ni^II^ ion is coordinated by two N and two O atoms from the tetra­dentate Schiff base ligand in a distorted square geometry. The crystal packing exhibits short inter­molecular Ni⋯Ni distances of 3.273 (3) Å.

## Related literature

For related structures, see: Ali *et al.* (2010 ▶); Hernandez-Molina *et al.* (1997 ▶); Niu *et al.* (2009 ▶); Radha *et al.* (1985 ▶).
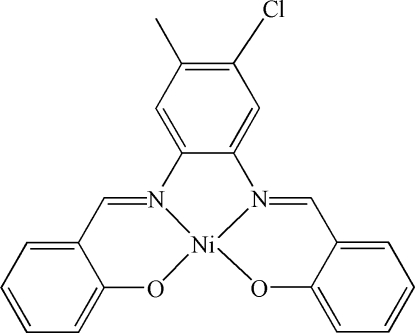


## Experimental

### 

#### Crystal data

[Ni(C_21_H_15_ClN_2_O_2_)]
*M* *_r_* = 421.51
Monoclinic, 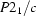

*a* = 11.0451 (10) Å
*b* = 8.0202 (7) Å
*c* = 19.5959 (17) Å
β = 106.37°
*V* = 1665.5 (3) Å^3^
*Z* = 4
Mo *K*α radiation
μ = 1.35 mm^−1^
*T* = 293 K
0.34 × 0.29 × 0.23 mm

#### Data collection

Bruker APEXII CCD area-detector diffractometer
Absorption correction: multi-scan (*SADABS*; Sheldrick, 2007 ▶) *T*_min_ = 0.658, *T*_max_ = 0.747
7946 measured reflections
2917 independent reflections
2155 reflections with *I* > 2σ(*I*)
*R*_int_ = 0.040

#### Refinement

*R*[*F* ^2^ > 2σ(*F* ^2^)] = 0.052
*wR*(*F* ^2^) = 0.153
*S* = 1.03
2917 reflections
245 parameters
H-atom parameters constrained
Δρ_max_ = 0.67 e Å^−3^
Δρ_min_ = −0.91 e Å^−3^

### 

Data collection: *APEX2* (Bruker, 2004 ▶); cell refinement: *SAINT-Plus* (Bruker, 2001 ▶); data reduction: *SAINT-Plus*; program(s) used to solve structure: *SHELXS97* (Sheldrick, 2008 ▶); program(s) used to refine structure: *SHELXL97* (Sheldrick, 2008 ▶); molecular graphics: *SHELXTL* (Sheldrick, 2008 ▶); software used to prepare material for publication: *SHELXTL*.

## Supplementary Material

Crystal structure: contains datablocks I, global. DOI: 10.1107/S1600536810046088/cv2792sup1.cif

Structure factors: contains datablocks I. DOI: 10.1107/S1600536810046088/cv2792Isup2.hkl

Additional supplementary materials:  crystallographic information; 3D view; checkCIF report

## Figures and Tables

**Fig. 1.**
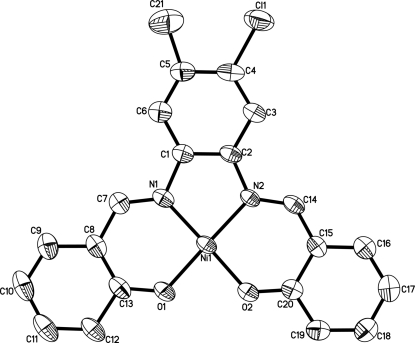
The molecular structure of the title complex with the atom-labelling scheme. Displacement ellipsoids are drawn at the 30% probability level. H atoms omitted for clarity.

## supplementary crystallographic information

### Comment

Schiff-base ligands, due to their excellent coordination ability, have been widely introduced into the coordination chemistry. Here, we report a new nickel complex based on a tetradentate Schiff-base ligand.

In the title compound (Fig. 1), the whole molecule is essentially planar with the mean deviation 0.0523 Å from the plane formed by all non-hydrogen atoms. The Ni^II^ ion is four-coordinated by two N atoms and two O atoms of the Schiff base ligand. The Ni—O and Ni—N bond lengths are all consistent with those found in other reported tetradentate Schiff base Ni complexes (Ali, *et al.*, 2010; Hernandez-Molina, *et al.*, 1997; Niu, *et al.*, 2009; Radha, *et al.*, 1985).

### Experimental

The synthesis of the title complex was carried out by reaction of Ni(ClO_4_)_2_.6H_2_O and the Schiff-base ligand with the molar ratio 1:1 in methanol under the stirring condition at room temperature. The filtrated solution was left to slowly evaperate in air to obtain single-crystal suitable for X-ray diffraction with the yield about 56%.

### Refinement

C-bound H atoms were placed in idealized positions with C—H distances of 0.93 and 0.96 Å, and were refined as riding atoms, with *U*_iso_(H) = 1.2-1.5 *U*_eq_(C).

### Crystal data

**Table:**

[Ni(C_21_H_15_ClN_2_O_2_)]	*F*(000) = 864
*M**_r_* = 421.51	*D*_x_ = 1.681 Mg m^−^^3^
Monoclinic, *P*2_1_/*c*	Mo *K*α radiation, λ = 0.71073 Å
Hall symbol: -P 2ybc	Cell parameters from 2314 reflections
*a* = 11.0451 (10) Å	θ = 2.5–27.0°
*b* = 8.0202 (7) Å	µ = 1.35 mm^−^^1^
*c* = 19.5959 (17) Å	*T* = 293 K
β = 106.37°	Block, red-brown
*V* = 1665.5 (3) Å^3^	0.34 × 0.29 × 0.23 mm
*Z* = 4	

### Data collection

**Table:**

Bruker APEXII CCD area-detector diffractometer	2917 independent reflections
Radiation source: fine-focus sealed tube	2155 reflections with *I* > 2σ(*I*)
graphite	*R*_int_ = 0.040
φ and ω scans	θ_max_ = 25.0°, θ_min_ = 1.9°
Absorption correction: multi-scan (*SADABS*; Sheldrick, 2007)	*h* = −13→13
*T*_min_ = 0.658, *T*_max_ = 0.747	*k* = −9→9
7946 measured reflections	*l* = −23→19

### Refinement

**Table:**

Refinement on *F*^2^	Primary atom site location: structure-invariant direct methods
Least-squares matrix: full	Secondary atom site location: difference Fourier map
*R*[*F*^2^ > 2σ(*F*^2^)] = 0.052	Hydrogen site location: inferred from neighbouring sites
*wR*(*F*^2^) = 0.153	H-atom parameters constrained
*S* = 1.02	*w* = 1/[σ^2^(*F*_o_^2^) + (0.1*P*)^2^] where *P* = (*F*_o_^2^ + 2*F*_c_^2^)/3
2917 reflections	(Δ/σ)_max_ = 0.018
245 parameters	Δρ_max_ = 0.67 e Å^−^^3^
0 restraints	Δρ_min_ = −0.91 e Å^−^^3^

### Special details

**Table:**

Geometry. All e.s.d.'s (except the e.s.d. in the dihedral angle between two l.s. planes) are estimated using the full covariance matrix. The cell e.s.d.'s are taken into account individually in the estimation of e.s.d.'s in distances, angles and torsion angles; correlations between e.s.d.'s in cell parameters are only used when they are defined by crystal symmetry. An approximate (isotropic) treatment of cell e.s.d.'s is used for estimating e.s.d.'s involving l.s. planes.
Refinement. Refinement of *F*^2^ against ALL reflections. The weighted *R*-factor *wR* and goodness of fit *S* are based on *F*^2^, conventional *R*-factors *R* are based on *F*, with *F* set to zero for negative *F*^2^. The threshold expression of *F*^2^ > σ(*F*^2^) is used only for calculating *R*-factors(gt) *etc*. and is not relevant to the choice of reflections for refinement. *R*-factors based on *F*^2^ are statistically about twice as large as those based on *F*, and *R*- factors based on ALL data will be even larger.

### Fractional atomic coordinates and isotropic or equivalent isotropic displacement parameters (Å^2^)

**Table:**

	*x*	*y*	*z*	*U*_iso_*/*U*_eq_	
Ni1	0.45347 (5)	0.83377 (6)	0.03397 (2)	0.0490 (2)	
Cl1	0.13615 (17)	0.8003 (2)	−0.31318 (8)	0.1129 (6)	
O1	0.4706 (3)	0.9080 (4)	0.12480 (13)	0.0619 (7)	
O2	0.6066 (3)	0.7335 (4)	0.07528 (14)	0.0582 (7)	
N1	0.2996 (3)	0.9374 (4)	−0.00538 (16)	0.0520 (8)	
N2	0.4388 (3)	0.7559 (4)	−0.05774 (15)	0.0471 (8)	
C1	0.2514 (4)	0.9118 (5)	−0.0800 (2)	0.0541 (10)	
C2	0.3286 (4)	0.8106 (5)	−0.1080 (2)	0.0531 (10)	
C3	0.2928 (4)	0.7764 (6)	−0.1805 (2)	0.0627 (11)	
H3	0.3430	0.7089	−0.1999	0.075*	
C4	0.1837 (5)	0.8422 (6)	−0.2230 (2)	0.0732 (14)	
C5	0.1058 (5)	0.9433 (6)	−0.1956 (3)	0.0721 (13)	
C6	0.1415 (4)	0.9765 (6)	−0.1252 (2)	0.0689 (12)	
H6	0.0908	1.0449	−0.1065	0.083*	
C7	0.2348 (4)	1.0221 (5)	0.0289 (2)	0.0583 (11)	
H7	0.1561	1.0618	0.0031	0.070*	
C8	0.2758 (4)	1.0584 (5)	0.1032 (2)	0.0606 (11)	
C9	0.1979 (6)	1.1596 (6)	0.1325 (3)	0.0792 (15)	
H9	0.1203	1.1955	0.1035	0.095*	
C10	0.2337 (7)	1.2052 (7)	0.2016 (3)	0.0926 (18)	
H10	0.1801	1.2683	0.2204	0.111*	
C11	0.3516 (7)	1.1567 (7)	0.2444 (3)	0.0880 (18)	
H11	0.3779	1.1922	0.2915	0.106*	
C12	0.4299 (5)	1.0572 (6)	0.2186 (2)	0.0756 (14)	
H12	0.5079	1.0253	0.2484	0.091*	
C13	0.3926 (5)	1.0026 (5)	0.1465 (2)	0.0598 (11)	
C14	0.5194 (4)	0.6572 (5)	−0.0743 (2)	0.0530 (10)	
H14	0.4997	0.6228	−0.1215	0.064*	
C15	0.6325 (4)	0.5970 (5)	−0.0291 (2)	0.0529 (10)	
C16	0.7113 (5)	0.4956 (5)	−0.0562 (3)	0.0669 (12)	
H16	0.6855	0.4652	−0.1039	0.080*	
C17	0.8255 (5)	0.4398 (6)	−0.0143 (3)	0.0774 (14)	
H17	0.8756	0.3714	−0.0334	0.093*	
C18	0.8657 (5)	0.4859 (6)	0.0564 (3)	0.0752 (13)	
H18	0.9440	0.4507	0.0847	0.090*	
C19	0.7894 (4)	0.5848 (6)	0.0855 (2)	0.0676 (12)	
H19	0.8173	0.6124	0.1335	0.081*	
C20	0.6729 (4)	0.6436 (5)	0.0451 (2)	0.0529 (10)	
C21	0.0008 (6)	1.0115 (12)	−0.2558 (4)	0.132 (3)	
H21A	−0.0784	0.9956	−0.2455	0.199*	
H21B	−0.0005	0.9539	−0.2989	0.199*	
H21C	0.0144	1.1283	−0.2613	0.199*	

### Atomic displacement parameters (Å^2^)

**Table:**

	*U*^11^	*U*^22^	*U*^33^	*U*^12^	*U*^13^	*U*^23^
Ni1	0.0643 (4)	0.0489 (4)	0.0354 (3)	−0.0117 (2)	0.0164 (2)	−0.0002 (2)
Cl1	0.1186 (13)	0.1466 (16)	0.0594 (9)	−0.0027 (10)	0.0020 (8)	−0.0031 (8)
O1	0.081 (2)	0.0689 (19)	0.0377 (15)	−0.0039 (16)	0.0190 (14)	−0.0014 (14)
O2	0.0714 (19)	0.0603 (17)	0.0423 (15)	−0.0052 (15)	0.0147 (14)	−0.0016 (13)
N1	0.072 (2)	0.0443 (18)	0.0400 (17)	−0.0150 (16)	0.0156 (15)	0.0010 (14)
N2	0.063 (2)	0.0462 (18)	0.0342 (16)	−0.0094 (16)	0.0174 (15)	0.0023 (14)
C1	0.062 (3)	0.051 (2)	0.049 (2)	−0.011 (2)	0.014 (2)	0.0046 (19)
C2	0.065 (3)	0.052 (2)	0.040 (2)	−0.0162 (19)	0.0116 (19)	0.0027 (17)
C3	0.076 (3)	0.065 (3)	0.046 (2)	−0.012 (2)	0.016 (2)	−0.003 (2)
C4	0.079 (3)	0.079 (3)	0.050 (3)	−0.019 (3)	−0.001 (2)	0.013 (2)
C5	0.074 (3)	0.071 (3)	0.062 (3)	−0.007 (3)	0.003 (2)	0.004 (2)
C6	0.073 (3)	0.065 (3)	0.066 (3)	−0.008 (2)	0.016 (2)	0.003 (2)
C7	0.070 (3)	0.049 (2)	0.061 (3)	−0.010 (2)	0.026 (2)	0.001 (2)
C8	0.086 (3)	0.048 (2)	0.057 (3)	−0.013 (2)	0.035 (2)	−0.001 (2)
C9	0.103 (4)	0.065 (3)	0.082 (4)	0.001 (3)	0.047 (3)	−0.002 (2)
C10	0.142 (6)	0.073 (3)	0.085 (4)	−0.008 (4)	0.068 (4)	−0.017 (3)
C11	0.141 (5)	0.077 (4)	0.061 (3)	−0.028 (3)	0.053 (4)	−0.017 (3)
C12	0.111 (4)	0.075 (3)	0.047 (2)	−0.019 (3)	0.035 (3)	−0.008 (2)
C13	0.090 (3)	0.050 (2)	0.046 (2)	−0.021 (2)	0.032 (2)	−0.0019 (19)
C14	0.075 (3)	0.047 (2)	0.039 (2)	−0.016 (2)	0.019 (2)	0.0006 (17)
C15	0.067 (3)	0.044 (2)	0.053 (2)	−0.0125 (19)	0.027 (2)	0.0010 (18)
C16	0.084 (3)	0.055 (3)	0.065 (3)	−0.007 (2)	0.028 (3)	0.005 (2)
C17	0.090 (4)	0.059 (3)	0.094 (4)	−0.004 (3)	0.044 (3)	0.003 (3)
C18	0.069 (3)	0.069 (3)	0.088 (4)	−0.004 (2)	0.022 (3)	0.009 (3)
C19	0.071 (3)	0.069 (3)	0.060 (3)	−0.012 (2)	0.013 (2)	0.005 (2)
C20	0.064 (3)	0.044 (2)	0.053 (2)	−0.0135 (19)	0.020 (2)	0.0092 (18)
C21	0.099 (5)	0.161 (8)	0.116 (5)	−0.012 (4)	−0.004 (4)	0.020 (4)

### Geometric parameters (Å, °)

**Table:**

Ni1—O1	1.835 (3)	C9—C10	1.349 (8)
Ni1—O2	1.841 (3)	C9—H9	0.9300
Ni1—N1	1.854 (3)	C10—C11	1.391 (8)
Ni1—N2	1.866 (3)	C10—H10	0.9300
Cl1—C4	1.729 (5)	C11—C12	1.375 (7)
O1—C13	1.306 (5)	C11—H11	0.9300
O2—C20	1.285 (5)	C12—C13	1.425 (6)
N1—C7	1.302 (5)	C12—H12	0.9300
N1—C1	1.422 (5)	C14—C15	1.398 (6)
N2—C14	1.298 (5)	C14—H14	0.9300
N2—C2	1.403 (5)	C15—C16	1.400 (6)
C1—C6	1.386 (6)	C15—C20	1.444 (6)
C1—C2	1.397 (6)	C16—C17	1.372 (7)
C2—C3	1.390 (6)	C16—H16	0.9300
C3—C4	1.363 (6)	C17—C18	1.381 (7)
C3—H3	0.9300	C17—H17	0.9300
C4—C5	1.395 (7)	C18—C19	1.390 (7)
C5—C6	1.349 (6)	C18—H18	0.9300
C5—C21	1.505 (8)	C19—C20	1.390 (6)
C6—H6	0.9300	C19—H19	0.9300
C7—C8	1.428 (6)	C21—H21A	0.9600
C7—H7	0.9300	C21—H21B	0.9600
C8—C13	1.402 (6)	C21—H21C	0.9600
C8—C9	1.417 (6)		
			
O1—Ni1—O2	83.34 (13)	C8—C9—H9	119.3
O1—Ni1—N1	95.17 (14)	C9—C10—C11	119.3 (5)
O2—Ni1—N1	178.50 (13)	C9—C10—H10	120.3
O1—Ni1—N2	178.89 (14)	C11—C10—H10	120.3
O2—Ni1—N2	95.55 (14)	C12—C11—C10	121.3 (5)
N1—Ni1—N2	85.94 (14)	C12—C11—H11	119.4
C13—O1—Ni1	127.3 (3)	C10—C11—H11	119.4
C20—O2—Ni1	127.9 (3)	C11—C12—C13	120.5 (5)
C7—N1—C1	120.3 (4)	C11—C12—H12	119.7
C7—N1—Ni1	126.3 (3)	C13—C12—H12	119.7
C1—N1—Ni1	113.4 (3)	O1—C13—C8	124.6 (4)
C14—N2—C2	122.4 (3)	O1—C13—C12	117.9 (5)
C14—N2—Ni1	124.4 (3)	C8—C13—C12	117.5 (4)
C2—N2—Ni1	113.2 (3)	N2—C14—C15	127.2 (4)
C6—C1—C2	119.2 (4)	N2—C14—H14	116.4
C6—C1—N1	127.6 (4)	C15—C14—H14	116.4
C2—C1—N1	113.2 (4)	C14—C15—C16	120.0 (4)
C3—C2—C1	119.1 (4)	C14—C15—C20	121.0 (4)
C3—C2—N2	126.6 (4)	C16—C15—C20	118.9 (4)
C1—C2—N2	114.3 (3)	C17—C16—C15	121.8 (5)
C4—C3—C2	119.8 (5)	C17—C16—H16	119.1
C4—C3—H3	120.1	C15—C16—H16	119.1
C2—C3—H3	120.1	C16—C17—C18	119.6 (5)
C3—C4—C5	121.6 (4)	C16—C17—H17	120.2
C3—C4—Cl1	120.7 (4)	C18—C17—H17	120.2
C5—C4—Cl1	117.6 (4)	C17—C18—C19	120.2 (5)
C6—C5—C4	118.3 (5)	C17—C18—H18	119.9
C6—C5—C21	131.9 (6)	C19—C18—H18	119.9
C4—C5—C21	109.4 (5)	C18—C19—C20	122.0 (5)
C5—C6—C1	122.1 (5)	C18—C19—H19	119.0
C5—C6—H6	119.0	C20—C19—H19	119.0
C1—C6—H6	119.0	O2—C20—C19	119.0 (4)
N1—C7—C8	124.8 (4)	O2—C20—C15	123.6 (4)
N1—C7—H7	117.6	C19—C20—C15	117.4 (4)
C8—C7—H7	117.6	C5—C21—H21A	109.5
C13—C8—C9	119.9 (4)	C5—C21—H21B	109.5
C13—C8—C7	121.6 (4)	H21A—C21—H21B	109.5
C9—C8—C7	118.4 (5)	C5—C21—H21C	109.5
C10—C9—C8	121.4 (6)	H21A—C21—H21C	109.5
C10—C9—H9	119.3	H21B—C21—H21C	109.5
